# Parental insights about supports for children with disabilities who are restricted eaters

**DOI:** 10.1111/1440-1630.70085

**Published:** 2026-04-12

**Authors:** Rene Fraser, Kim Bulkeley, Rebecca Barton

**Affiliations:** ^1^ The University of Sydney Camperdown New South Wales Australia

**Keywords:** capacity building, eating, family‐centred practice, fussy eating, parents, picky eating, responsive approaches

## Abstract

**Introduction:**

Restricted eating—where children eat less than 30 foods, eliminate whole food groups, or avoid mealtimes—has far‐reaching consequences beyond nutrition. Due to this complexity, parents may seek help from multiple health professionals. Although literature reports on various supports, there is limited understanding of parents' experiences. This research aims to explore parents' perspectives on supports provided to children with disabilities who have been identified as restricted eaters.

**Methods:**

A constructivist qualitative design with reflexive inductive thematic analysis of semi‐structured interviews with seven parents of children with restricted eating was employed.

**Consumer and Community Involvement:**

There was no consumer or community involvement in this study.

**Findings:**

Parents described factors impacting access to services and a variety of supports that can be described on two axes: (a) focus of support and (b) responsiveness of support. Parents highlighted that supports on the more responsive end of the continuum—where clinicians acknowledged parental expertise, adapted strategies to individual child needs, and attended to family context—were perceived as more helpful. Conversely, rigid or prescriptive approaches were often experienced as misaligned with their child's needs, increasing parental stress and reducing perceived usefulness.

**Conclusion:**

The findings of this study highlight a need to critically appraise standardised structured approaches. Recommendations have been made to support shifts in practice towards services that are family‐centred, focussed on capacity building, and promote responsive approaches to mealtimes.

## PLAIN LANGUAGE SUMMARY

Many children with disabilities have trouble eating. They may only eat a small number of foods or avoid entire food groups like vegetables or meat. When children eat less than 30 foods or do not eat food from all the food groups, this is called restricted eating. For children with disabilities, this often lasts longer than other children and can affect growth and how bodies work. These children may find it hard to eat with others and avoid family meals, missing out on important family time. Although we know about therapies to help these children, we do not know much about what parents think about these therapies. Understanding parents' views is important because they play a crucial role in children's lives. We interviewed seven parents about their children with disabilities who are restricted eaters. We asked about the therapy their children received and recorded everything they told us. We carefully analysed every word to understand parents' experiences. Parents described two approaches: Some therapists worked directly with children, whereas others worked with parents. They also told us about two different styles—some therapists used standard programmes for all children, whereas others created personalised plans for each child's specific needs. Our findings suggest that personalised approaches may work better than one‐size‐fits‐all rigidly structured programmes. We recommend therapists work closely with families to develop supports that fit each child's unique needs and family situation. This study provides important guidance to help therapists, children, and families to work together to support children's eating.

**Key Points for Occupational Therapy**

- Occupational therapists should consider tailored, family‐centred eating support rather than one‐size‐fits‐all programmes.
- Occupational therapists should build parent capacity to understand their child's eating.
- Occupational therapists should consider moving away from developmental models towards a focus on meaningful mealtime participation for children and families.

## 1 INTRODUCTION

Family mealtimes are a formative part of family routines and relationship building (Hosokawa et al., 2023). Participation in family mealtimes and eating is influenced by factors within the child, their social and physical environments, and routines and tasks associated with eating; hence, occupational therapists are well placed to provide support in this area (Chilman et al., 2021; Wolstenholme et al., 2020). Restricted eating—where a child eats less than 30 foods and eliminates whole food groups or has difficulty participating in mealtimes—has implications beyond growth and nutrition, including impacting family meals and participation in social events involving food (Aponte et al., 2019; Connor et al., 2023; Simione et al., 2020). This definition is used to operationalise the variety of descriptors that are used in this area of practice while maintaining a focus on function and observable actions. In this study, we did not rely on access to a formal diagnosis, for example, paediatric feeding disorder (PFD). Although we acknowledge the benefits of uniform terminology and clear diagnostic criteria, focussing on a formal diagnosis would limit understanding the experience of a large number of parents whose children experience difficulties with eating and participating in mealtimes but do not have a formal diagnosis. This is particularly relevant for diagnoses such as PFD, which was not widely used in Australia at the time of this study.

Child, parent, relationship, and contextual factors may influence the restricted eating of children with disabilities, and parents often seek help from health professionals, including occupational therapists, speech pathologists, and dietitians (Chilman et al., 2023). Recent research indicates that the most frequent initiator of referral to these services is parents; however, little is known about parents' experiences of these services (Chilman et al., 2023).

A previous scoping review (Fraser et al., 2023) describes a variety of approaches to service delivery, including relational approaches that support parents to respond to their child's emotional and physical needs thoughtfully and consistently and strict standardised procedures from manualised programmes (Black & Aboud, 2011). These approaches include behaviour‐based supports, food play and exposure, supports focussed on child skill development, and those that consider the impact of the task and the environment (Aponte et al., 2019; Chawner et al., 2019; Chilman et al., 2023; Hladik et al., 2024; Scahill et al., 2023). Supports may be provided by therapists through individual child‐focussed sessions, group sessions, parent–child dyads, parent training, and parent education (Chawner et al., 2019; Chilman et al., 2023; Scahill et al., 2023; Thorsteinsdottir et al., 2021). Parent training approaches were described as teaching parents to implement specifically structured programmes and parent education, which is a broader approach to increase knowledge around managing eating and mealtimes (Scahill et al., 2023).

Despite the wide array of therapeutic support approaches, most are reported as effective (Chawner et al., 2019), but how effectiveness is measured is a multifaceted consideration. To date, there is a lack of in‐depth understanding of parents' experience of these supports and outcomes. Volume of food consumed is the most frequent outcome measure, followed by food variety (Chawner et al., 2019; Fraser et al., 2023). However, consideration of broader factors, including relationship and autonomy, has not been reported in the current evidence base. Concerns have been raised about a focus on increases in volume and variety of foods as a primary outcome measure. A more diverse food intake can be achieved in a meaningful and child‐affirming way and should not overshadow the importance of the parent–child relationship or compromise a child's autonomy (Rowell et al., 2023). Less frequently used outcome measures, including decreases in behaviours of concern and the ability to participate in mealtimes, are more closely aligned with these broader factors, highlighting the need for appraising current approaches to outcome measurements (Aponte et al., 2019; Chawner et al., 2019).

The effectiveness and appropriateness of traditional child‐only‐focussed supports are being questioned as they are often centred around the achievement of typical milestones, where it is assumed there is a need to intervene when developmental progression is different (Barfoot et al., 2024). This philosophy is embedded in developmentalism and entwined with ableist assumptions about who decides what is acceptable development (Mosleh & Gibson, 2022). In the context of mealtimes, most supports are focussed solely on either the child or the parent, with few specifically focussed on strengthening their relationship (Fraser et al., 2023). Yet it is acknowledged that when working with all children, there should be a focus on protecting and strengthening the parent–child relationship, building capacity within the family unit, adopting strengths‐based approaches, and maintaining the child's dignity and rights (Early Childhood Intervention Australia, 2015).

Relationship‐focussed supports aim to strengthen parent–child engagement, enabling positive interactions and supporting the unique needs of the child (Barfoot et al., 2024). A relational approach may encourage less focus on goals aimed at approximating developmental norms (Mosleh & Gibson, 2022) and shift the focus to prioritise the parent–child relationship (Barfoot et al., 2024) while ensuring a child is consuming enough nutrients to nourish their body; supporting a child's autonomy and confidence to explore new foods; and enabling participation in food‐related family events. This requires us to re‐appraise our understanding of what constitutes a successful family mealtime (Sanchez, 2024). When the focus is shifted to participation in family events, including mealtimes, occupational therapists are well placed to provide tailored supports that align with the occupational therapy process and practice (Chilman et al., 2023).

Parents of children with disabilities are likely to have tried a range of strategies to support their child before seeking professional help (Bernheimer & Weisner, 2007). When parents of children with disabilities who are restricted eaters do seek support, significant time is spent locating and accessing appropriate services (Winston, 2015). Even when appropriate services are engaged, parents continue to face access barriers, including travel time to appointments and financial and logistical impacts on daily family activities (Raatz et al., 2021). Considering these factors, it is likely that parents will have high expectations of appointments. Parents value therapists who are available, willing to work collaboratively, professional, knowledgeable, have strong interpersonal skills, and focus on goal achievement (Fairweather et al., 2022; Fingerhut et al., 2013; Kolehmainen et al., 2010; Simione et al., 2020; Wong Chung et al., 2020). Many of these traits are aligned with family‐centred supports. It is widely acknowledged that the provision of family‐centred supports that increase parent self‐efficacy is associated with high‐quality practice and is positively received by parents (Almasri et al., 2018; Kruijsen‐Terpstra et al., 2014). Family‐centred supports involve therapists responding to the lead of families to develop individualised supports with meaningful outcomes that support long‐term sustainable change (Bourke‐Taylor, 2017; Fingerhut et al., 2013).

Although an understanding of parent perspectives is essential for determining if services are family‐centred and responsive to their needs, few studies have explored the perspectives of parents of children with disabilities who are restricted eaters in relation to the supports they have received. The studies that have included parent perspectives focussed on mixed child populations without specific attention to parents of children with disabilities (Simione et al., 2020) or focussed on parental perceptions of a specific programme (Hladik et al., 2024). Within the context of interdisciplinary supports, occupational therapists need to understand the perspectives of parents in order to deliver high‐quality, family‐centred services (Franklin & Rodger, 2003). This research aims to explore parents' experiences of supports provided to children with disabilities who have been identified as restricted eaters.

## 2 METHODS

Ethical approval for this study was gained from the University of Sydney (Protocol Number 2021/540).

### 2.1 Positionality statement

The authors position themselves as occupational therapists with many years of combined experience. All authors approach this research from a rights‐based perspective informed by their professional experiences. The authors have a shared commitment to family‐centred practice and critical disability perspectives.

### 2.2 Research approach

We employed a qualitative research design informed by a relativist ontological stance aligned with a constructivist epistemological approach, which enabled the authors to explore and gain insight into the subjective experiences of participants while simultaneously acknowledging the role of the research team in knowledge creation (Braun & Clarke, 2022). Data were collected using semi‐structured interviews and analysed using reflexive thematic analysis (Braun & Clarke, 2022). Throughout, the authors actively reflected on their positionality, questioning themselves and each other, leading to rich data analysis supporting rigour and confirmability (Braun & Clarke, 2022). This design enabled a thorough exploration of the perceptions and experiences of parents of children with disabilities and restricted eating, acknowledging that individuals will create their own understandings based on their diverse, complex experiences (Creswell & Creswell, 2023).

### 2.3 Study context and recruitment

Participants in this study were self‐identified parents of children with disabilities who are restricted eaters, as defined in Section 1. Purposive snowball sampling was used to support the recruitment of participants (Johnson et al., 2020). Eligibility criteria are displayed in Table 1.

**TABLE 1:** Eligibility criteria.

Criteria
Parents of a child who
Is aged between 0 and 18 years
Has a lifelong permanent disability (NDIS, 2025)
Is a restricted eater (eats less than 30 foods and excludes whole food groups or participation in mealtimes is affected)
Is not exclusively bottle‐fed or breastfed
Parents who have received support for their child's restricted eating in the last 5 years

Recruitment was completed between November 2021 and August 2023. Information about the study was provided to services, Australia‐wide, identified through the National Disability Insurance Scheme (NDIS) providers list that supports this group of children, asking them to share a recruitment flyer with families. In addition, recruitment information was posted on Australian public disability‐related groups on Facebook, encouraging broader sharing about the study with other interested people. Potential participants were invited to contact the research team to register their interest. They were sent a participant information statement and invited to ask questions before confirming participation.

### 2.4 Data collection

The first author completed in‐depth, semi‐structured interviews with participants via Zoom, ensuring that a textured and granular description of each individual's experience was gained (Trainor & Bundon, 2021). An interview schedule (available on request), informed by literature, included open‐ended questions that explored the parents' experience of services designed to address their child's restricted eating. The interview was piloted by the first author with an experienced researcher and clinician who provided feedback.

Recruitment, data collection, and data analysis were completed simultaneously. Ten people, all who met the eligibility criteria, initially responded to recruitment by emailing the research team; however, only seven subsequently replied to arrange interviews. Six of the seven parent interviews occurred with a mother, and one interview occurred with both parents. At the start of each interview (lasting between 30 and 60 minutes), verbal consent was gained. Immediately after the interviews, the first author completed field notes, supporting researcher reflexivity (Trainor & Bundon, 2021).

### 2.5 Data analysis

**FIGURE 1:**
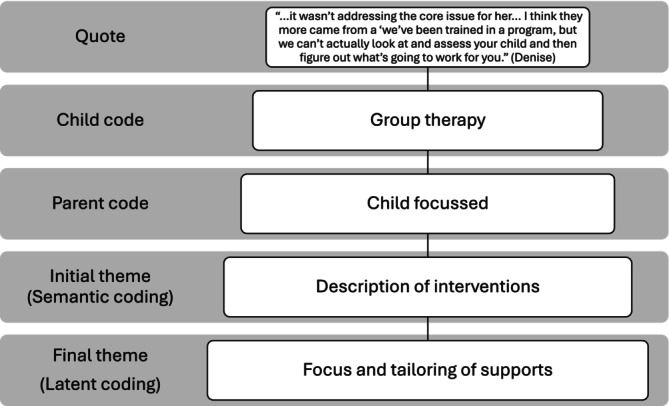
Theme development.

Recordings were stored securely, transcribed, and deidentified by assigning pseudonyms to all participants and removing identifying information. Transcriptions were verified by the first author and participants, ensuring accuracy (Braun & Clarke, 2022). Deidentified transcripts were uploaded to NVivo 12, supporting organisation and analysis of data. The authors adopted an iterative approach, moving fluidly between data collection and analysis, including coding and theme identification, to determine when enough data had been collected to enable a rich and complex exploration of the research question (Braun & Clarke, 2021). The first author read all transcripts for familiarity and then applied detailed codes. Codes were then examined by returning to the interviews. Through discussion with all three authors, codes were combined into initial themes, for example, an aggregation of parent education and group therapy codes into a theme of ‘description of interventions’. As coding moved from semantic to latent analysis, themes were further refined by all three authors using mind maps, word clouds, and robust discussion to identify shared meaning and combine codes into final themes (Figure 1) related to the research question, representing a coherent story about the data (Braun & Clarke, 2022).

## 3 FINDINGS

All seven parents (Table 2) self‐identified their children as restricted eaters, describing their child's eating in the following ways: a restricted variety and volume of food, eating only specific brands, sensitivity to texture, difficulties joining in eating with the rest of the family, and reduced participation in events involving food. All parents lived in Australia, with five living in regional locations and two living in metropolitan areas.

**TABLE 2:** Demographic information about participants.

Pseudonym	Age of child/children (years)	Household composition	Diagnosis	Location	Types of services accessed
Elizabeth and Dave	12 and 4	2 parents and 3 children	Autism spectrum disorder (ASD)	Regional, New South Wales (NSW)	Speech pathology and occupational therapy
Nathalia	13	1 parent and 1 child	Rare neurodevelopmental condition	Metropolitan, NSW	Occupational therapy
Kate	7	2 parents and 3 children	Rare neurodevelopmental condition	Regional, Victoria	Dietetics and speech pathology
Ella	12	2 parents and 3 children	ASD, Down syndrome, and multi‐sensory impairments	Regional, NSW	Speech pathology, occupational therapy, dietetics, and paediatrician
Denise	8	2 parents and 3 children	Attention‐deficit hyperactivity disorder (ADHD) and Ehlers–Danlos syndrome	Regional, NSW	Speech pathology, occupational therapy, dietetics, and paediatrician
Sheryl	8	2 parents and 3 children	ASD, ADHD, oppositional defiant disorder, and anxiety	Regional, NSW	Speech pathology, occupational therapy, and dietetics
Alice	5	1 parent and 1 child	ASD and avoidant/restrictive food intake disorder (ARFID)	Metropolitan, NSW	Speech pathology, occupational therapy, and dietetics

Findings will be reported in relation to three key themes: (1) access to services, (2) focus and tailoring of supports, and (3) impact of professionals on the success of supports.

### 3.1 Access to services

Access to services, including the types of services and challenges associated with them, was described by all participants and provided a context of resource scarcity. Parents described accessing a range of professional supports (e.g., occupational therapy, speech pathology, and dietitians) through face‐to‐face and telepractice. Barriers to access identified by all participants included absence of services in certain geographical areas, services without therapists with the required skills and experience, and long waiting lists. Associated with these factors, some parents reported driving long distances to access services: ‘… we did [a structured program] with a therapist, which was over an hour drive from here.’ (Ella). Staff turnover was identified as a barrier to accessing services: ‘… we've had two come and go … and then back on the waitlist again … it's really impossible to get assistance.’ (Alice).

Telepractice was described as an option for overcoming some barriers to accessing services, providing timely access to consistent, experienced professionals. Some parents reported that engaging in telepractice was difficult, whereas for others, it was successful:

Fred had no interaction with her. She would just come on board with me [online] … I'd meet her once a month … she helped to try to give me ideas to strengthen what I was doing at home and keep me on track. (Sheryl)

### 3.2 Focus and tailoring of supports

Participants provided information on the focus of the supports they received and the way standardised and tailored approaches were used. Nuance and variation were evident within the categories presented below, with parents describing supports that shifted within and between categories at different points in time.

#### 3.2.1 Child‐focussed standardised supports

The majority of parents described participating in structured standardised programmes that require professionals to complete specific training, delivering support according to guidelines. Despite professionals being qualified to deliver these programmes, parents identified that some lacked the required knowledge and specific expertise: ‘… there's a lot of … lack of understanding about the area’ (Denise). Parents often entered these programmes with hope and high expectations: ‘… when we found out, we were like, “Oh, this is it. This is the thing.” ’ (Ella).

Many parents described these services as rigid and not well matched to their child and family; for example, ‘… it is so rigid … the expectation is you will sit and you will stay … for an ADHD kid is quite overwhelming’ (Sheryl). Elizabeth further highlighted a mismatch between services and family priorities, explaining: ‘… having to play, exaggerate, smoosh food up … I do it, but in the back of my head, I've got a billion other things that I'm thinking. I find it very hard to be in the moment during food therapy.’

Denise highlighted the lack of tailoring of programmes:

… it wasn't addressing the core issue for her … I think they more came from a ‘we've been trained in a program, but we can't actually look at and assess your child and then figure out what's going to work for you’.

Some parents explained that their child experienced high levels of distress during these programmes:

… it got to a point where Fred would throw chairs at [the therapist]… Spit, scream …

… [it] was actually quite traumatic … Fred was literally like … a cat, trying to scratch and claw at the door and wall to get out with high‐pitched screams. (Sheryl)

Furthermore, some parents identified that their child's emotional safety was not prioritised; for example, ‘… if you don't feel safe around food, you can't scaffold … when your reactions are fear‐based reactions’ (Denise).

Some parents reached a point where they identified that these programmes were not meeting their child's needs. Despite identifying that it was causing distress and gains were not being transferred to home, some parents took a break and sought out the programme with a different provider, whereas others described feeling conflicted about ceasing supports: ‘I ended up saying I think I'm torturing this child … I know that there is research … I feel like we're torturing him.’ (Sheryl).

Some parents reported not seeing meaningful change in what their child ate: ‘… tolerating it [food] in the room and touching it and having it on their mouth … he'll do all of that. He will not put it in his mouth.’ (Ella). When parents identified positive changes, they were described as lacking meaning for the family or taking a very long time to achieve: ‘It took me … three till she was seven … to go from Vegemite toast to a Vegemite toasted sandwich’ (Denise). Standardised programmes were described as expensive and time‐consuming due to the amount of food preparation required.

[the therapist] will give me a list of what she wants me to buy … it's expensive as well …. I had to buy all new bowls and plates, because they needed to be all white … It's a good 40 minutes of prep. (Elizabeth)

#### 3.2.2 Child‐focussed tailored supports

Most participants reflected on supports that were focussed on the child and tailored to meet the child's and family's needs, including modifications to the environment and adaptation of the task, alongside skill development in the child.

Families described success with modifying the environment to support their child's participation in family meals:

… little things like … a scallop chair … to help with his posture … and now we have a spot … here at the dining table and that's actually brought Fred to the table …. The fact that he's now sitting at the table, probably 50% of the time and eating is massive. (Sheryl)

Another parent described how their child had been supported to develop skills with eating using adapted utensils and food, allowing them to participate in a variety of family meals:

… if we go out and about, we just take a lunch pack sort of thing with his bowls. We've sort of got it down to a fine art. He's got a bowl with his food in it and we'll have the spoons in there … And he'll feed himself with the spoon …. (Ella)

Yet Nathalia described how individual tailored support focussed on skill development in a clinic environment had not transferred to the home environment: ‘I tried to replicate the pizza, but I don't have … a special pizza oven and it just doesn't quite work the same ….’

#### 3.2.3 Parent‐focussed standardised supports

A few parents described a variety of parent education resources, including information booklets and education as an adjunct to therapy or, in one instance, a parent choosing to attend a professional course to gain more information. At times, parents identified these as helpful as described by Ella: ‘… that stuff was helpful and I found that good. I found that there was some good things in there.’ Other examples were provided where education was not useful as it was not tailored to the child and family: ‘… in an ideal world, I know that we should all be eating dinner on a dining room table, with placemat settings. I have been taught all of that … Unfortunately, I can't implement that … I am making between three and four different dinners a day …’ (Elizabeth).

Denise described being provided with generic advice that was not helpful: ‘… it wasn't helpful for her. It might have been helpful for another child.’

#### 3.2.4 Parent‐focussed tailored supports

Some participants described supports where the professional worked with parents developing strategies to support their child with eating and mealtimes. Parents valued the professionals' time spent collaborating with them and sharing relevant knowledge, developing strategies to support their child. Sheryl explained, ‘… there's lots of liaising with me about what he is and isn't eating, giving me the ideas of how I can introduce stuff differently’. Parents frequently identified that it was difficult to transfer strategies to the home environment, valuing support from professionals to implement strategies within the daily routine: ‘… whilst it puts a lot more pressure on me to do stuff at home, that's where we've had more success.’ (Sheryl).

This theme—emphasising focus and tailoring of supports—represents a spectrum described by parents, which can be mapped along two continua: from parent‐focussed to child‐focussed and from tailored to standardised approaches that can be developed in a nuanced and individualised way.

### 3.3 Impact of professionals on the success of supports

Parents identified that the approach adopted by individual professionals impacted the success of supports, with parents describing those who work in a family‐centred manner more positively. Parents appreciated professionals acknowledging their effective strategies: ‘When people have the chat and acknowledge that some of the stuff you're doing anyway is really good … that's helpful … it gives you a bit of a spring in your step, fills you up a bit’ (Kate).

However, parents also described advice from professionals that was not respectful of their concerns.

I had a paediatrician say to me, ‘What does it matter if she only eats hot chips when you go out?’ And I was like … in that one scenario it doesn't but that's all she eats … at home too. (Denise)

Some parents described situations where the skill and approach of an individual therapist impacted the success of a standard programme.

It was very, very, very unhelpful. And I don't know that it was the actual program itself. I think it was the therapist. We've seen lots and lots of therapists over the years and I think there was just a really bad mismatch in terms of personality and that kind of thing. She just didn't read his cues …. (Ella)

## 4 DISCUSSION

**FIGURE 2:**
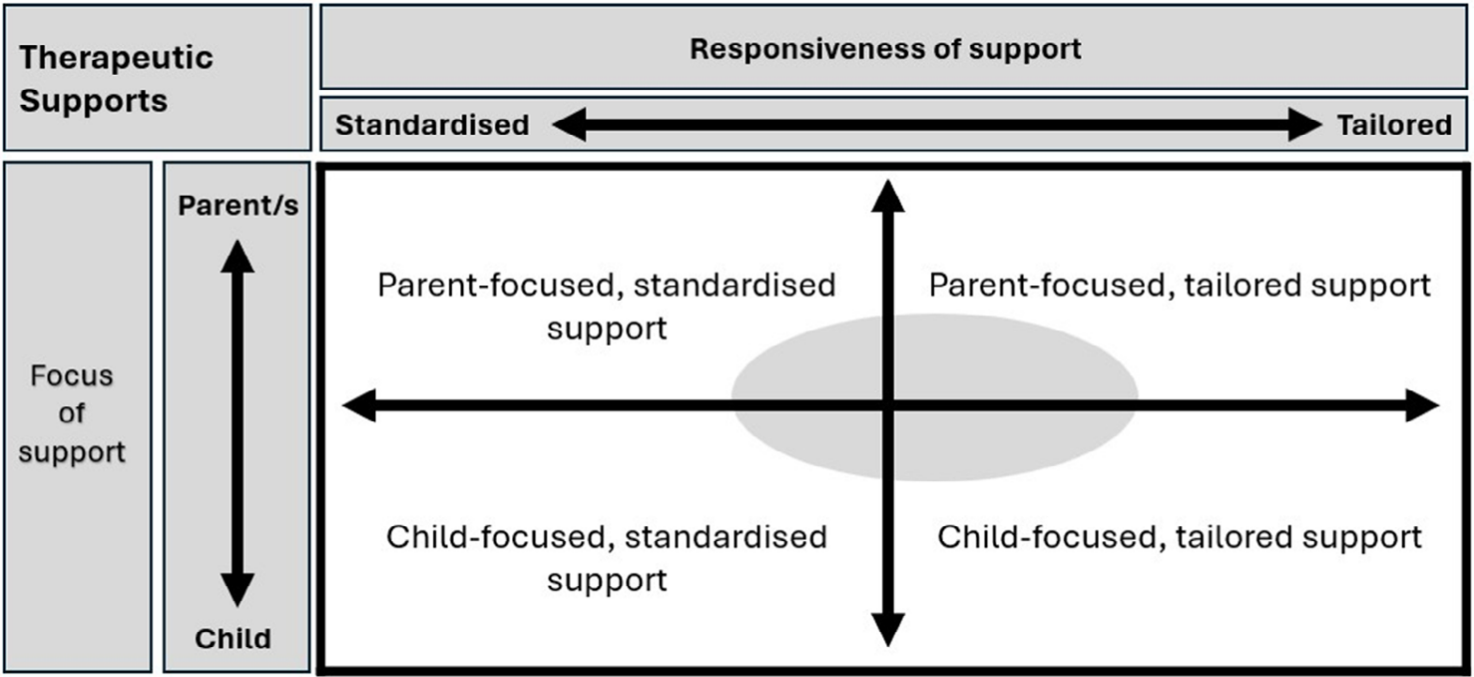
Positioning therapeutic supports: mapping focus and responsiveness.

This research aimed to explore parents' experiences of services for their children with disabilities who are restricted eaters. The results describe a varied focus (parent or child) and responsiveness (standardised or tailored) of supports. High‐quality practice guidelines emphasise working collaboratively with families to determine how best to meet their needs (Early Childhood Intervention Australia, 2015), encouraging professionals to move fluidly between services focussed on the child and their environment and those focussed on the parent (Johnsson & Bulkeley, 2024). The results of this study suggest that the supports parents accessed can be conceptualised as existing along two continua (Figure 2). The vertical axis reflects the focus of support, and the horizontal axis reflects the degree of responsiveness. The lower end of the vertical axis represents supports that are solely focussed on the child, with supports aimed at addressing problems residing in the child. The top of the vertical axis represents supports aimed solely at parents to provide education, develop tailored individualised strategies, and build capacity within the family unit. The horizontal responsiveness axis represents supports that were delivered in strict adherence to standardised programmes on the left, moving to individualised, flexible, and tailored supports aimed at supporting children in natural environments on the right of the axis. The shaded oval represents an area where therapists shift their focus and degree of responsiveness fluidly in relation to client need, drawing elements from a range of approaches. This study highlights the need to consider if standardised programmes delivered to a strict protocol can meet the needs of families of children with complex disabilities who are restricted eaters. Positive descriptions in the current study involve professionals engaging with families, developing tailored, individualised strategies that support the child and family unit. Tailored and flexible approaches focussed on the family are well placed to ensure that parents can embrace their child with a disability, understand their child to better meet their needs, and consider goals for their child that do not necessarily conform to a narrow definition of success (Lee et al., 2023).

Our findings align with literature on positive parent–therapist relationships, emphasising the value parents place on therapist availability, collaboration, expertise, and goal achievement (Fairweather et al., 2022; Kolehmainen et al., 2010; Simione et al., 2020). Many parents in the current study described experiences with professionals who did not validate their concerns, support collaborative working relationships, or have adequate knowledge and expertise. This highlights a discord between practice and the literature. Family‐centred practice is consistently identified in high‐quality guidelines (Imms et al., 2024; Johnsson & Bulkeley, 2024), but not consistently described by this group of parents. Unfortunately, at times, this resulted in negative experiences for both the child and parent. When working with families, professionals must be aware of the possible negative impacts of support and reflect and adapt service provision accordingly (Grandisson et al., 2023).

Raatz et al. (2023) identified that many professionals report low levels of confidence when working with children with eating issues. The greater use of standardised programmes and protocols is potentially linked to the low levels of confidence that novice clinicians (Unsworth, 2001) experience when supporting children with eating restrictions. Despite the initial appeal of these standardised programmes, we identified examples where the development of family capacity was not supported. In these instances, the experiences of families were largely not aligned with family‐centred practice (Fingerhut et al., 2013). Chilman et al. (2023) have also identified concerns with these programmes related to the large commitment required from families, lack of evidence, and the resource‐intensive training required to deliver them.

Parents in the current study identified that the approach of individual professionals impacted the success of supports, with parents describing professionals who work in a family‐centred manner as fostering parental efficacy. Family‐centred approaches recognise parental expertise and enable parents and children to be heard and see the possibilities of what they can do (Dunn, 2017).

The current study indicates that family capacity is positively influenced by the provision of timely supports. Strategies, including telepractice and parent education (not parent training), may support the provision of timely supports with consistent experienced therapists (Grandisson et al., 2023; Hladik et al., 2023). Parent education was identified as helpful, but further support was needed to implement and tailor the strategies to the context. Tailored supports increase the responsiveness to families' needs, positively impacting capacity building within the family unit (Grandisson et al., 2023). Professionals must consider the need to ask permission to share information and provide information that specifically meets the child's and family's needs in order to provide respectful and responsive supports (Graham et al., 2021).

Parents spoke positively of individualised tailored supports that considered the environment and task as much as addressing skill development of the individual child. This approach reflects the principles of occupational therapy (Law et al., 1996), including broader constructs of task and environmental support to increase participation for children and their families (Fingerhut et al., 2013; Rowell et al., 2023). When therapists consider broader constructs and collaboratively problem‐solve with parents, they position the eating issues in a context that contributes positively to parental self‐efficacy, strengthening the parent–child relationship (Barfoot et al., 2024). Child‐focused supports lacking collaboration risk missing opportunities for parent‐child problem‐solving and relationship strengthening (Barfoot et al., 2024).

When therapy was reported as solely focussed on the child with a disability, we noted a risk that support will be underpinned by ableist beliefs and achievement of standardised developmental milestones without recognition of contextual factors (Barfoot et al., 2024; Gerlach & McFadden, 2022). This developmentalist approach focuses on perceived deficits at the level of the individual child (Reeves et al., 2022), which are less effective than supports that are individualised and contextualised (Barfoot et al., 2024).

## 5 LIMITATIONS AND FUTURE RESEARCH

The findings of the current study highlight that parents need support that prioritises their child's safety and is responsive to the needs of their child and family. This study is a deep dive into the experiences of parents of children with disabilities who are restricted eaters using a qualitative design and a small sample size and highlights emerging factors for this population. To document diverse parental experiences, this study had minimal exclusion criteria and used a broad definition of restricted eating, which may limit the applicability of these findings to the specific population with more specific diagnoses around childhood eating issues. A larger quantitative approach focussing on specific diagnostic groups and age ranges may be of benefit in understanding parents' experiences, including an efficacy study on responsive approaches in relation to mealtimes. The perspectives of children with restricted eating are an area that warrants attention in future research.

## 6 CONCLUSION

This study described the types of supports provided to children with disabilities who are restricted eaters and their families. Parents identified barriers to accessing supports, including missing services, long waiting lists, and lack of availability of therapists with required skills and expertise. Parents identified supports varying in focus (child to parent) and responsiveness (Figure 1). This framework, derived from lived experiences, may assist in guiding the development of supports for children and young people with restricted eating and disabilities. Parents indicated they may benefit from clinicians who demonstrate responsiveness to both child and family needs, suggesting the potential value of tailored supports, founded on collaborative relationships where parental insights and voices are respected. It is important to consider if a shift in practice away from rigid developmentalism to a focus on capacity building for meaningful participation, with consideration of a broader framing of behaviours and positive outcomes, is required. Supports aimed at enabling parents to understand their child's eating in a manner that is defined by them and not by rigid externally imposed expectations may support responsiveness and contribute to family capacity building. Training to support therapists and a commitment from organisations and broader service systems may support a continued shift towards the provision of individualised, family‐centred services that have a focus on capacity building.

## AUTHOR CONTRIBUTIONS

This research was completed as part of Rene Fraser's higher degree by research with support from her supervisors, Kim Bulkeley and Rebecca Barton.

**Rene Fraser:** Conceptualization (lead); data curation; formal analysis (lead); investigation; methodology (lead); project administration; visualization; writing—original draft preparation. **Kim Bulkeley:** Conceptualization (supporting); formal analysis (supporting); methodology (supporting); writing—review and editing. **Rebecca Barton:** Conceptualization (supporting); formal analysis (supporting); methodology (supporting); writing—review and editing.

All authors approved the final manuscript.

## CONFLICT OF INTEREST STATEMENT

The authors declare no conflicts of interest.

## DECLARATION OF USE OF ARTIFICIAL INTELLIGENCE

Select sentences were entered into Microsoft Copilot to support grammatical accuracy, including the use of active voice.

## Data Availability

The datasets generated and analysed during the current study are not publicly available and are only available to the research team.
